# Synergistic Coupling of Intrinsic Internal Electric Field and Macroscopic Polarization in a Photocatalytic Fuel Cell for Efficient Antibiotic Degradation

**DOI:** 10.3390/nano16060354

**Published:** 2026-03-13

**Authors:** Xicheng Li, Bicheng Ji, Jiajie Bao, Jiuwei Wu, Changzheng Wang

**Affiliations:** 1 Institute of Advanced Materials, Beijing Key Laboratory of Functional Materials for Building Structure and Environment Remediation, School of Environment and Energy Engineering, Beijing University of Civil Engineering and Architecture, Beijing 100044, China; lecter_lee@163.com (X.L.); bichengji@163.com (B.J.); baojiajie2505@163.com (J.B.); 2 Department of Nuclear Technology and Application, China Institute of Atomic Energy, Beijing 102413, China; 3 Engineering Practice Innovation Center, Beijing University of Civil Engineering and Architecture, Beijing 102616, China

**Keywords:** photocatalytic fuel cell, TiO_2_ nanotube array, photoanode, internal electric field, emerging pollutants

## Abstract

The concurrent challenges of environmental pollution and energy scarcity necessitate advanced sustainable technologies. Photocatalytic fuel cells (PFCs) offer a promising route by coupling pollutant degradation with energy recovery. However, the synergistic interplay between anode intrinsic properties and macroscopic polarization effects remains inadequately understood. Herein, a BiOBr-doped TiO_2_ nanotube array photoanode with engineered oxygen vacancies was developed to construct a synergistically enhanced PFC system. XPS, EPR, and DFT analyses confirm the formation of oxygen vacancies and favorable band bending, inducing an internal electric field that markedly promotes charge separation and interfacial reaction kinetics. As a result, the charge separation efficiency is enhanced by approximately fourfold relative to pristine TiO_2_ nanotube arrays. Under the combined action of the internal electric field and self-bias-induced polarization field, photogenerated electrons and holes undergo directional transport and effective utilization. The optimized PFC achieves 78% sulfamethoxazole degradation within 180 min, representing a 1.38-fold improvement. Degradation pathways and toxicity evolution were further elucidated using LC–MS and Fukui function analysis, highlighting the critical role of electric field-driven charge regulation in high-performance PFCs.

## 1. Introduction

Antibiotic drugs are an important weapon for combating diseases and are widely used worldwide. Studies have shown that these drugs cannot be completely metabolized after being ingested by humans or livestock, and a large amount of the drugs enter the environment through excretion, posing a huge potential risk to ecological stability [1,2,3]. The performance of conventional water treatment processes in dealing with these new pollutants is unsatisfactory. Moreover, although membrane technology has good treatment efficiency in removing new pollutants, its high-energy-consumption defect makes it unable to effectively match the current low-carbon demand [4]. Advanced oxidation methods have made significant progress in the field of organic pollutant degradation, among which photocatalysis has attracted much attention due to its easily accessible driving energy and high degradation efficiency [5,6,7]. The core of photocatalysis lies in the intrinsic properties of the catalyst, and many excellent photocatalysts have been developed. However, it must be pointed out that the development and application of photocatalysis have been constrained by the problem of insufficient carrier separation efficiency.

Many strategies have been developed by people to improve the carrier separation efficiency of catalysts, such as doping elements, constructing heterojunctions, and external field assistance [8,9,10,11]. The photocatalytic fuel cell (PFC) is an important extension of photocatalysis, which is different from ordinary external field assistance. PFC uses the self-bias voltage between the two electrodes to form macro polarization, enhancing the separation efficiency of photogenerated carriers [12,13,14]. This method has been applied in the field of removing pollutants from water, as in our previous work, using in situ-grown classical catalyst TiO_2_ nanoparticles on a foam nickel substrate, clearly explaining the key role of macro polarization in the PFC system in the performance of ciprofloxacin in water [15]. It must be pointed out that, although PFC significantly improves the reaction efficiency compared to traditional photocatalysis, the intrinsic properties of the photocatalyst still need to be further optimized. Specifically, the transfer rate of photogenerated charges in the bulk of the photoanode catalyst and the overall efficiency of actual participation in the interface reaction have not received sufficient attention in the development of the PFC system. Further in-depth exploration of the intrinsic optimization of the photoanode catalyst is of crucial scientific significance and application value for the future development of this system.

In this study, a composite photoanode catalyst was constructed by introducing oxygen vacancy-modified BiOBr onto TiO_2_ nanotube arrays (TNAs). The resulting material exhibited a charge separation efficiency nearly four times higher than that of pristine TNAs. This pronounced enhancement is primarily attributed to the formation of an internal electric field, which effectively facilitates the separation and directional transport of photogenerated charge carriers. Simultaneously, under the macroscopic polarization field induced by self-bias, photogenerated electrons are efficiently transferred to the cathode to participate in reduction reactions, while photogenerated holes are retained at the anode. Moreover, the reduction in the surface depletion layer enables a greater fraction of photogenerated holes to engage in interfacial reactions. To evaluate the degradation performance of the modified photoanode within the PFC system, the representative antibiotic sulfamethoxazole (SMX) was selected as a model pollutant. The enhanced degradation behavior and underlying mechanisms were systematically investigated using a combination of electron spin resonance (ESR), liquid chromatography–mass spectrometry (LC–MS), and density functional theory (DFT) calculations. This work highlights the critical role of optimizing the intrinsic charge transport properties of photocatalysts in PFC systems and advances the fundamental understanding of PFC operating mechanisms from the perspective of charge transport dynamics.

## 2. Materials and Methods

### 2.1. Chemicals and Materials

All chemical reagents used in this work were of analytical grade and used without further purification. Ammonium fluoride (NH_4_F), sulfamethoxazole (C_10_H_11_N_3_O_3_S), bismuth nitrate pentahydrate (Bi(NO_3_)_3_·5H_2_O) and sodium bromide (NaBr) were purchased from Macklin Biochemical Co., Ltd. (Shanghai, China). Anhydrous ethanol (C_2_H_6_O_2_), glycerol (C_3_H_8_O_3_) and sodium hydroxide (NaOH) were purchased from Beijing Tong Guang Fine Chemicals Company (Beijing, China). Hydrofluoric Acid (HF) was purchased from Xilong Scientific Co., Ltd. (Guangzhou, China). Nitric acid (HNO_3_) was purchased from China National Pharmaceutical Group Co., Ltd. (Beijing, China).

### 2.2. The Synthesis of BiOBr/TiO_2_ Composite Nanotube Array

The photoanode was prepared via a facile two-step synthesis procedure. First, TiO_2_ nanotube arrays were fabricated by anodic oxidation. Specifically, 1.224 g of NH_4_F was dissolved in a mixed solvent of propylene glycol and deionized water (volume ratio 17:3) to serve as the electrolyte. Pre-treated titanium foil was immersed in the electrolyte, connected as the anode to a DC stabilized power supply, while a platinum electrode was used as the cathode. The anodization process was conducted for 6 h. After anodization, the obtained TiO_2_ nanotube array substrates were thoroughly rinsed with anhydrous ethanol and deionized water, naturally air-dried, and subsequently annealed in a muffle furnace at 600 °C with a heating rate of 5 °C min^−1^ for 30 min.

In the second step, BiOBr was deposited onto the TiO_2_ nanotube arrays through a chemical bath deposition method under water-bath heating at 40 °C. X mmol (X = 1, 2, and 3) of Bi(NO_3_)_3_·5H_2_O was dissolved in 20 mL of 0.1 mol L^−1^ mannitol solution to form solution A. Meanwhile, 1 mmol of NaBr was dissolved in 20 mL of deionized water to provide the bromide source and was designated as solution B. Both solutions were maintained at 40 °C prior to use. The TiO_2_ nanotube array substrates were alternately immersed in solutions A and B for 2 min each. After each immersion, the substrates were rinsed with anhydrous ethanol to prevent blockage of the nanotube openings by excessive BiOBr deposition. The BiOBr loading was regulated by adjusting the Bi precursor concentration in solution A. The resulting samples prepared with 1, 2, and 3 mmol of Bi precursor were denoted as BTNA-1, BTNA-2, and BTNA-3, respectively.

### 2.3. Characterization

X-ray diffraction (XRD) patterns of different samples were recorded using the XRD-6100 (XRD; Shimadzu, Kyoto, Japan). The morphology and surface composition were observed using a scanning electron microscope (ZEISS Gemini SEM 360, Zeiss, Jena, Germany). The morphology and composition of this sample were explored using transmission electron microscopy (TEM, TEOL H7650, Hitachi, Tokyo, Japan). EDS data were obtained from TEM analysis for elemental content analysis. The chemical states of the samples were measured by using X-ray photoelectron spectroscopy (XPS, Thermo ESCALAB250Xi, Thermo Fisher Scientific, Waltham, MA, USA), and the binding energy of the C 1s peak at 284.8 eV was taken as an internal standard. Electron paramagnetic resonance (EPR) and electron spin resonance (ESR) spectra were collected with a Bruker A200 spectrometer (Bruker, Ettlingen, Germany). The absorbance properties of the photoanode materials were tested using UV–visible diffuse reflectance spectroscopy (DRS, Shimadzu UV-2600, Japan) with a measurement wavelength range of 200–800 nm. The electrochemical experiments were carried out with an electrochemical workstation (CHI660E electrochemical station, Shanghai Chenhua, Shanghai, China) using a three-electrode system.

### 2.4. Photoelectrochemical Measurements

Briefly, 5 mg of catalyst and 20 µL of Nafion solution were dispersed in 480 µL of deionized water. Then, 40 µL of this mixture was dropcast onto a fluorine-doped tin oxide (FTO) glass substrate (1 × 1 cm^2^) to serve as the working electrode. A Pt sheet and a saturated Ag/AgCl electrode were used as the counter and reference electrodes, respectively. Transient photocurrent responses, electrochemical impedance spectroscopy, and Mott–Schottky measurements were performed in 0.1 M Na_2_SO_4_ aqueous solution as the electrolyte. The photoanode and Pt cathode were connected by a wire, forming a closed circuit in the photocatalytic fuel cell system. The effective volume of the electrolytic cell was 50 mL, and the light source was irradiated from the side of the photoanode. Other details were consistent with those used in the photocatalytic degradation process. The J-V curves and power density curves were measured using a programmable resistor ranging from 1 Ω to 9 MΩ and a multimeter.

### 2.5. Explanation of the Experimental Setup and Procedures

In a typical photocatalytic degradation experiment, the initial concentration of the sulfamethoxazole (SMX) aqueous solution was set at 10 mg L^−1^ (C_0_ = 10 mg L^−1^). Prior to irradiation, the suspension was magnetically stirred in the dark to establish adsorption–desorption equilibrium between the catalyst surface and SMX molecules. After equilibrium was achieved, the photocatalytic reaction was initiated using a 300 W xenon lamp equipped with an AM 1.5 G filter (CEL-HXF300-T3, Education Au-light Co., Ltd., Beijing, China). All experiments were conducted at room temperature (25 °C). Except for pH-dependent studies, all degradation tests were performed at the initial pH of the solution. The residual SMX concentration during the reaction was monitored using ultraviolet–visible (UV–Vis) spectrophotometry at a characteristic wavelength of 260 nm. To minimize experimental error, all degradation experiments were carried out in triplicate, and the average values were used to construct degradation curves.

To investigate the influence of solution pH on catalytic performance, the initial pH of the SMX solution was adjusted using 0.1 M NaOH or 0.1 M HCl. To evaluate the effects of coexisting ions, 1 mL of a 10 mM inorganic ion solution was added to the SMX solution. For cation interference experiments, nitrate was used as the counter anion, whereas sodium ions (Na^+^) served as the counter cation in anion interference tests.

Radical scavenging experiments were performed by introducing isopropanol, p-phenylenediamine, and sodium oxalate as quenchers for hydroxyl radicals (·OH), superoxide radicals (·O_2_^−^), and photogenerated holes (h^+^), respectively, to identify the dominant reactive species involved in the degradation process.

For stability and reusability assessments, the catalyst was recovered after each reaction, thoroughly washed with deionized water and ethanol, dried at 60 °C, and subsequently reused in successive degradation cycles to evaluate its durability and recyclability.

### 2.6. DFT Calculations

The work functions of different photoanode materials were calculated based on first-principles theory, and the calculations of the Fukui function of pollutants were conducted using quantum chemistry software. For details of the calculations, please refer to the Supplementary Materials.

## 3. Results and Discussion

### 3.1. The Morphology and Structure of the BTNA Photoanode

**Figure 1:**
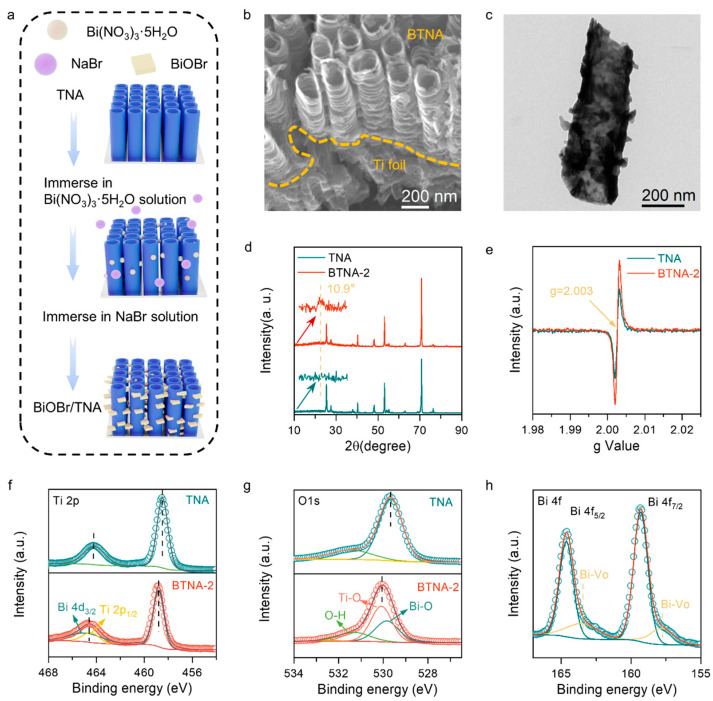
(**a**) Schematic diagram of the preparation process of BTNA photoanode. (**b**) SEM image and (**c**) TEM image of BTNA-2. The (**d**) XRD patterns and (**e**) EPR spectra of BTNA-2 and TNA. (**f**) Ti 2p, (**g**) O 1s and (**h**) Bi 4f high-resolution XPS spectrum of BTNA-2 and TNA.

The SEM images clearly show the specific morphology of BTNA-2 (Figure 1b and Figure S1a–c). BiOBr grows between and on the nanotubes, and the overall morphology of the composite catalyst approaches a “branch-and-leaves” pattern (Figure 1b and Figure S1b), effectively utilizing the excellent electron transport properties brought by the regular arrangement of TNA [16,17,18]. Additionally, through the image of SEM, the deposition of BiOBr on the surface of BTNA-1 is poor, and less BiOBr may not have a sufficient impact on the charge separation efficiency of the composite catalyst (Figure S1a). Excessive BiOBr deposition on BTNA-3 has basically covered the TiO_2_ nanotubes, affecting the overall light absorption capacity of the composite catalyst (Figure S1c). To further demonstrate the growth pattern of BiOBr on the nanotubes, TEM characterization was performed on the BTNA-2 sample (Figure 1c). The loading amount of BiOBr on the stripped nanotubes is slightly reduced compared to the SEM test, which may be due to the partial detachment of BiOBr grown on the nanotubes by ultrasound. However, it is worth noting that many BiOBr still adhere to the nanotubes, consistent with the previously designed growth pattern. Figure S1d shows the distribution of each element. Since TiO_2_ accounts for the largest proportion in the composite material and the nanotube array is grown on the Ti foil, the signal distribution of Ti element is the densest. The signal of O element is slightly weaker than that of Ti element, which is due to the presence of O element in both semiconductors that make up the composite material. The proportion of BiOBr in the composite material is low, so Bi element and Br element have the weakest signals, but their uniform distribution can still be observed on the composite material.

The phase analysis of the BTNA composite catalyst was carried out using XRD. The XRD patterns of the BTNA-2 and TNA samples are shown in Figure 1d. The crystal structure of the TNA substrate was not affected during the growth of BiOBr. The signals at 2θ = 25.4°, 38.0°, 48.0°, and 54.7° corresponded to the anatase phase TiO_2_ (PDF#21-1272), and the diffraction peaks were strong and sharp, indicating that the crystallization degree of TiO_2_ in the substrate was good. Different from TNA, the BTNA-2 sample had a weak diffraction peak at 10.9°, which corresponded to the (001) crystal plane of BiOBr (PDF#09-0393). However, due to the loading amount, the intensity of its characteristic peak was weak. Mannitol was used as a structural directing agent to make BiOBr grow in a planar manner. The thin crystal growth mode made the BiOBr surface rich in oxygen vacancies. To further confirm the oxygen vacancy situation, electron paramagnetic resonance (EPR) characterization was performed on BTNA-2 and ordinary TNA, and the results are shown in Figure 1e. The abundant oxygen vacancies provide a foundation for the modification of the photoanode to establish a stronger internal electric field and provide more active sites.

Subsequently, the surface elemental composition and valence states of the samples were investigated using XPS. The binding energy was calibrated using the C 1s peak at 284.8 eV. The Ti, Bi, and O elements in TNA and BTNA-2 can be clearly distinguished (Figure S1), consistent with the EDX elemental mapping results (Figure S2). The high-resolution Ti 2p spectra (Figure 1f) show characteristic doublet peaks, and a slight shift is observed in BTNA-2 compared to TNA, where the BTNA-2 sample can be deconvoluted at 464.6 eV, attributed to the Ti and Bi orbitals [19]. The high-resolution O 1s spectra (Figure 1g) display two peaks for TNA at 529.7 and 531.2 eV, attributed to lattice oxygen and surface hydroxyl groups, respectively. In BTNA-2, these peaks shift slightly toward higher binding energies (530.1 and 531.4 eV), suggesting a possible decrease in local electron density around oxygen species. Such shifts may be associated with interfacial charge redistribution after the formation of the BiOBr/TiO_2_ heterojunction. The Bi 4f high-resolution spectrum (Figure 1h) shows the characteristic Bi(III) 4f doublet at 157.7/163.3 eV. Notably, an additional low-binding-energy shoulder is observed in BTNA-2. This feature may be related to the presence of low-valent Bi species, potentially induced by oxygen vacancies (Bi–Vo) or changes in local coordination structure [20]. Combined with the subtle shift observed in the Ti 2p spectrum, these results are consistent with possible electronic interaction at the BiOBr/TiO_2_ interface and the formation of a spatial charge region.

### 3.2. The Mechanism by Which the Internal Electric Field Optimizes the Carrier Transport Behavior of BTNA-2

**Figure 2:**
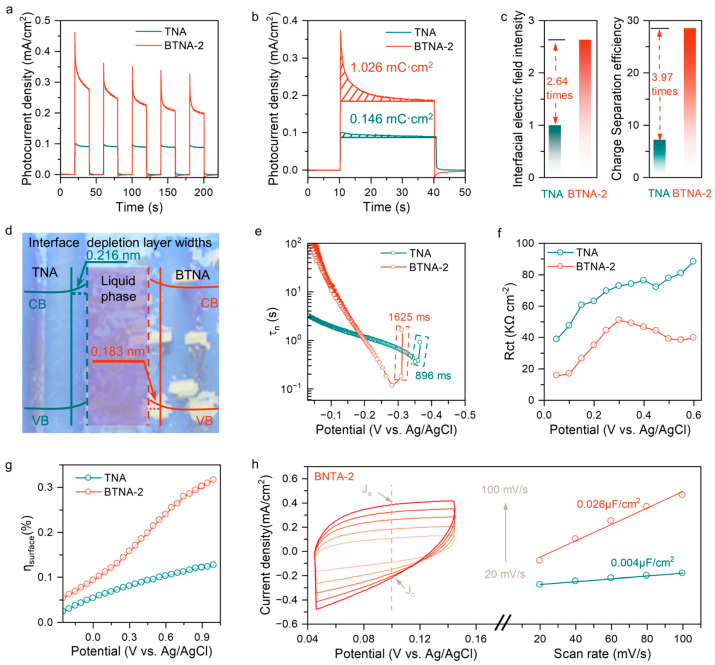
(**a**) Photocurrent responses showing enhanced charge separation in BTNA-2. (**b**) Photogenerated charge density. (**c**) BTNA-2 exhibits significantly enhanced internal electric field strength and charge separation efficiency. (**d**) Depletion layer widths, highlighting the structural advantages of BTNA-2. (**e**) BTNA-2 has a longer carrier lifetime, (**f**) lower R_ct_ value and (**g**) higher interface charge mobility than TNA. (**h**) BTNA-2 has a high electrochemical active area.

In order to deeply understand the internal electric field situation and charge transport properties of the anode catalyst, a series of electrochemical tests were conducted. As shown in Figure 2a, the photocurrent of BTNA-2 was significantly stronger than that of the unmodified TNA substrate under intermittent illumination, indicating that the formation of the TiO_2_/BiOBr heterojunction effectively drives the separation of photogenerated carriers in the bulk [21,22]. Additionally, the photogenerated charge density was measured using the transient photocurrent density (Figure 2b). According to existing research, by subtracting the steady-state photocurrent from the measured transient photocurrent density and integrating it over time, the internal electric field is proportional to the accumulated charge on the surface area [23]. The results show that the photogenerated charge densities of BTNA-2 and TNA are 1.026 mC/cm^2^ and 0.146 mC/cm^2^, respectively, and the internal electric field intensity has increased by 2.64 times (Equation (S1), Figure 2c). To more specifically characterize the key role of the internal electric field in driving the separation of carrier pairs in the composite catalyst, Na_2_SO_3_ electrolyte was used to capture the holes generated by the catalyst during illumination. By calculating the ratio of the maximum photocurrent to the actual photocurrent in the experiment, the carrier separation efficiency of the catalyst can be easily obtained (Equation (S2), Figure S3) [24]. Encouragingly, the carrier separation efficiency of BTNA-2 is 3.97 times higher than that of TNA (Figure 2c).

The aforementioned experiments confirmed that the establishment of the Ti-O-Bi charge transport channel significantly inhibited the recombination of photogenerated carriers. The charge transport behavior was then studied. The Mott–Schottky plot clarified the influence of loading BiOBr on the carrier concentration and depletion layer of the catalyst (Figure S4). The flat band potential of the composite catalyst shifted positively and moved towards the valence band, indicating that, in the Fermi-level alignment process, electrons in TiO_2_ flowed towards BiOBr, and the significant increase in carrier concentration could be attributed to oxygen vacancies, which acted as electron donors and improved the charge transport efficiency. This observation result was consistent with the XPS results. The depletion layer width (W_b_) of BTNA-2 slightly decreased, possibly due to the space charge region near the contact interface, where the energy band curved downward as electrons continuously accumulated during the flow towards BiOBr, and holes tended to anchor on TiO_2_ (Equation (S3), Figure 2d) [25].

We compared the OCP attenuation curves under light and dark conditions to study the surface carrier transport characteristics in the BTNA-2 photoanode. Figure S5 shows that, compared with TNA, the OCP decay rate of BTNA-2 is significantly reduced, indicating that the photogenerated charge carriers are effectively separated under the influence of the internal electric field. The carrier lifetime (*τ*_n_) can be used to compare the charge recombination rate at the electrode/electrolyte interface [26]. Obviously, after the light stops, the *τ*n observed on the TNA photoanode is 896 milliseconds (Figure 2e), while the *τ*n value of BTNA-2 is 1625 milliseconds, and the significantly increased photogenerated carrier transient lifetime reflects the excellent role of the Ti-O-Bi charge transport channel in the internal electric field. Impedance testing revealed the efficiency of charge migration within the catalyst’s bulk and at the interface. The slightly increased bulk impedance further confirmed the successful preparation of the heterojunction. Although these heterojunction interfaces are beneficial for charge separation, they may also introduce additional interface barriers or defect states in certain cases, resulting in a higher resistance for the charge transmission at these interfaces [27]. The interface transmission resistance and bulk resistance of BTNA-2 were much lower than those of TNA, which facilitated the participation of photogenerated carriers in the interface reaction (Figure S6). The C_ss_ values derived from the EIS test indicated that charges were transferred through the surface states to the electrolyte. The abundant surface states of BTNA-2 were conducive to the participation of photogenerated charges in the interface reaction, which was consistent with the significant enhancement of degradation performance (Figure S7). Figure 2f clearly shows that the interface transmission resistance of BTNA-2 was significantly lower than that of TNA under different voltage conditions. The “η_surface_” represents the proportion of holes successfully injected into the water oxidation layer at the interface. It can be calculated based on the LSV data under different conditions (Figure S8) [28]. In Figure 2g, the maximum value of η_surface_ for TNA was only 15%, while for BTNA-2 it increased to 32%. This result is very close to the previous charge separation efficiency test results.

In addition, CV tests were used to characterize the electrochemical active area of the catalyst to demonstrate the excellent properties of the composite material. The results showed that, after the modification with defect-type BiOBr, the active sites of BTNA-2 were significantly more than those of the ordinary TNA (Figure 2h and Figure S9). This phenomenon supported that the carrier separation efficiency had been greatly enhanced. Combined with the aforementioned characterization results, it can be explained that, under the action of the internal electric field, the charges were directly migrated to the BiOBr phase, while the photogenerated holes remained in TNA. This spatial separation characteristic enabled more carriers to participate in the interface reaction, making the catalyst have more reaction active sites. In conclusion, a series of electrochemical test results indicated that the charge transport of BTNA-2 was significantly improved under the influence of the Ti-O-Bi channel, which will bring foreseeable enhancement to the performance of the PFC system in degrading organic pollutants in water.

### 3.3. The BTNA-2 Photoanode Catalyst in the PFC System Exhibits Enhanced SMX Degradation Efficiency and Electricity Generation Performance

**Figure 3:**
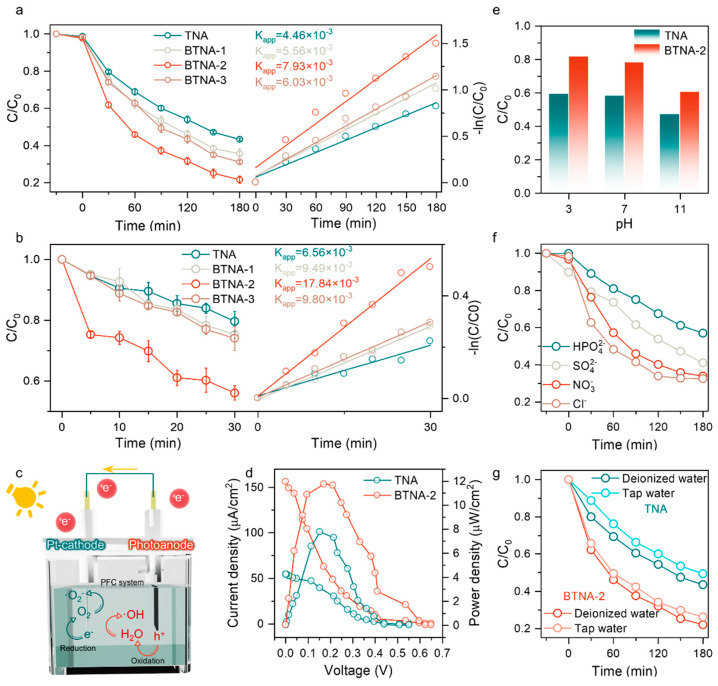
Catalytic degradation of SMX in photocatalytic fuel cell system: (**a**) 180 min and (**b**) 30 min. (**c**) Schematic diagram of the operation of photocatalytic fuel cell. (**d**) J-V curves and corresponding power densities of PFC based on TNA and BTNA-2. Effect of water quality conditions on SMX degradation: (**e**) pH, (**f**) coexisting inorganic ions and (**g**) different background solvents.

The prepared catalyst was applied to the photoanode of the PFC system. In the degradation of the model pollutant SMX, the dark adsorption effect of all samples was not strong, and similar results were achieved. It is notable that, after activation, BTNA-2 could reach a degradation rate of approximately 78% within a reaction period of 180 min, with a degradation rate much higher than TNA (Figure 3a). After fitting the degradation data, it was concluded that the degradation process basically followed first-order reaction kinetics and belonged to a photocatalytic reaction. As previously tested, although BTNA-1 and BTNA-3 still had better degradation performance for SMX than pure TNA, the mismatch in loading led to their performance being weaker than BTNA-2. Based on the degradation test, the rapid degradation of SMX within 30 min after activation can be observed. We further refined the sampling interval to verify the pathway of the catalytic reaction (Figure 3b). The faster decrease in the SMX concentration indicates that BTNA-2 has an efficient free radical generation efficiency, which is attributed to the strong driving force provided by the internal electric field for carrier separation. In the PFC system, the BTNA-2 photoanode plays a key role, promoting electrical energy output through the rapid charge transfer channel of Ti-O-Bi and the effect of the internal electric field. The carriers generated by the BTNA-2 photoanode absorbing photon energy are further separated under the action of the microscopic internal electric field, while the macroscopic polarization field formed by the connection of the external circuit transports electrons to the cathode for reduction reactions, and holes are anchored at the anode, oxidizing the pollutant directly or generating hydroxyl radicals through the oxidation of water molecules for the degradation reaction (Figure 3c). To evaluate their photoelectric performance, the PFC operation conditions of different photoanodes in SMX were tested. As shown in Figure 3d, BTNA-2 exhibited the best performance, with an open-circuit voltage of 0.65 V, a short-circuit current density of 0.15 mA/cm^2^, and a maximum output power density of approximately 12 μW/cm^2^.

Considering that reaction conditions in practical applications are substantially more complex than those of simulated pollutant solutions prepared under laboratory conditions, the effects of various environmental factors on degradation performance were systematically investigated. Evaluation of the influence of pH is essential, and the results indicate that both BTNA-2 and TNA exhibit good degradation stability under acidic and neutral conditions, whereas a slight decline in performance is observed under alkaline conditions (Figure 3e). It should be noted that the surface charge characteristics of TiO_2_ differ under acidic and alkaline environments. Under acidic conditions, the positively charged TiO_2_ surface favors the adsorption of negatively charged SMX molecules, thereby shortening the mass transfer distance and slightly enhancing the degradation efficiency. In contrast, under alkaline conditions, electrostatic repulsion between the TiO_2_ surface and SMX molecules hinders adsorption, resulting in a modest decrease in degradation performance.

In addition, inorganic anions commonly present in natural waters may also influence catalytic degradation processes. Therefore, the effects of four representative anions on the degradation performance of the composite photoanode were examined. The results show that, except for HPO_4_^2−^, the other three anions exerted only a minor influence on degradation efficiency (Figure 3f). The pronounced inhibitory effect of HPO_4_^2−^ is likely due to its adsorption onto the catalyst surface during the reaction, which passivates active catalytic sites and suppresses the reaction [29]. Notably, the presence of Cl^−^ resulted in the least reduction in degradation performance among the tested anions. This behavior can be attributed to the competitive interaction between Cl^−^ and highly oxidative photogenerated holes; however, Cl^−^ can be oxidized by holes to form ClO^−^ species with certain oxidative activity, thereby minimizing its overall inhibitory effect on the catalytic process [30,31].

Furthermore, to more closely simulate realistic water matrices, tap water was used to prepare the SMX solution for evaluating the catalyst performance under practical conditions. The results demonstrate that, at relatively low concentrations of various inorganic ions, the degradation of SMX in the PFC system is scarcely affected, with a performance decrease of only approximately 3% (Figure 3g). Five cycles of degradation experiments were conducted to evaluate the performance stability of the catalyst. The used BTNA-2 photoanode catalyst was washed with ethanol and deionized water to remove the residual contaminants and then dried for use in the next degradation process. The results are shown in Figure S10; after five cycles of photocatalytic degradation, only about 2.3% of the performance was lost, indicating that the BTNA-2 sample has excellent catalytic performance stability.

### 3.4. The Free Radical Generation Mechanism of BTNA-2 and the Degradation Pathway Analysis of SMX in the PFC System

**Figure 4:**
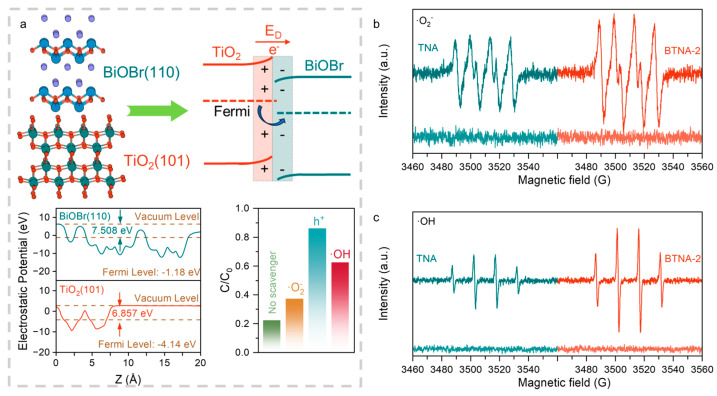
(**a**) Schematic diagram of charge transfer between TiO_2_ and BiOBr, as well as experiments on work function and radical quenching. ESR spectra of (**b**) ·O_2_^−^ and (**c**) ·OH.

To further establish the structure–activity relationship between the catalyst and the generation of reactive oxygen species based on the aforementioned characterizations, first-principles calculations and radical scavenging experiments were employed. Upon contact between BiOBr and TiO_2_, spontaneous Fermi level alignment occurs, driving intrinsic charge transfer from the phase with a lower work function to that with a higher work function. During this process, an internal electric field is spontaneously formed at the interface between the two phases (Figure 4a). As demonstrated in the preceding results, the presence of a low-valence shoulder peak in the high-resolution Bi 4f spectrum indicates that BiOBr receives electrons from TiO_2_, which is consistent with the work function calculations. Specifically, electrons are transferred from anatase TiO_2_ (work function: 6.857 eV) to BiOBr (7.508 eV). This finding provides strong evidence for the successful construction of an internal electric field, which supplies an electrostatic driving force for spatial charge separation, significantly prolongs carrier lifetimes, and enhances the driving force for interfacial reactions.

Radical scavenging experiments were conducted to identify the dominant reactive species involved in the photocatalytic degradation of SMX over the BTNA-2 photoanode. As shown in Figure 4a, the addition of sodium oxalate, a hole scavenger, significantly suppressed hole participation, resulting in a sharp decrease in degradation efficiency to only 13.9%. In contrast, the addition of p-benzoquinone and isopropanol caused relatively minor reductions in degradation efficiency, to 62.1% and 37.6%, respectively. These results indicate that h^+^ remains the dominant reactive species in the photocatalytic process, while ·O_2_^−^ and ·OH generated from photogenerated electrons cooperate with holes to promote SMX degradation.

Electron spin resonance (ESR) spectroscopy was further employed to detect the generation of hydroxyl radicals and superoxide radicals under light irradiation. DMPO was used as the spin-trapping agent for both species, and a xenon lamp equipped with a 400 nm cutoff filter served as the visible-light source. The ESR spectra shown in Figure 4b,c reveal that no characteristic signals of either radical were observed under dark conditions, whereas pronounced signals corresponding to hydroxyl and superoxide radicals emerged upon light irradiation. This confirms that both reactive species are generated by the composite material under illumination and that the composite effectively utilizes visible light, indicating an extended light absorption range. Furthermore, comparison of the ESR signal intensities indicates that the composite generates a greater number of reactive species than pristine TiO_2_ nanotube arrays, demonstrating that BiOBr coupling enhances electron–hole separation efficiency and visible-light responsiveness. Upon contact between the two phases, electrons tend to flow from TiO_2_ to BiOBr due to the difference in work functions. This results in the formation of a positively charged space region near the TiO_2_ side, arising from electron depletion, which induces an upward bending of the energy bands. Conversely, the accumulated electrons in BiOBr generate a negatively charged region at the interface, causing the energy bands to bend downward. Consequently, the internal electric field is directed from TiO_2_ to BiOBr, and the interfacial band structure exhibits an alignment resembling that of an S-type heterojunction. Under these conditions, photoexcited electrons in TiO_2_ are transported through the external circuit to the cathode to participate in the reaction, whereas the photogenerated electrons in BiOBr preferentially recombine with holes in TiO_2_. The synergistic effect of the microscopic internal electric field and the macroscopic polarization significantly enhances charge carrier separation, thereby improving the catalytic performance, which is consistent with the previously observed increase in photocurrent and the optimized interfacial charge behavior revealed by electrochemical characterization.

**Figure 5:**
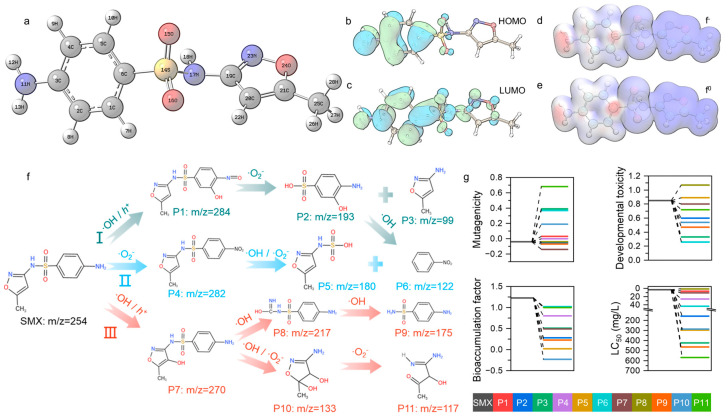
(**a**) The optimized SMX molecular structure model. (**b**) HOMO and (**c**) LUMO electron density distributions of SMX. Fukui function isosurface maps indicating (**d**) electrophilic (f^−^) and (**e**) radical (f^0^) reactive sites. (**f**) Proposed degradation pathway of SMX in photocatalytic fuel cell and (**g**) toxicity assessment of degradation intermediates.

To elucidate the degradation pathways of SMX, density functional theory (DFT) calculations were performed to determine the highest occupied molecular orbital (HOMO), lowest unoccupied molecular orbital (LUMO), and Fukui functions, thereby identifying the reaction-initiating sites. The optimized molecular structure of SMX is illustrated in Figure 5a. As shown in Figure 5b, the HOMO is mainly localized on the benzene ring and amino group, indicating that these regions act as electron-donating sites. In contrast, the LUMO is distributed over the entire molecular backbone, suggesting that these sites are more prone to electron acceptance. The Fukui function isosurface maps (Figure 5d,e) and condensed Fukui function values (Table S1 in the Supplementary Materials) reveal that both electrophilic and radical reactions predominantly occur at the C6 and N11 sites on the benzene ring. The high f^−^ and f^0^ values confirm that these positions are particularly susceptible to radical attacks from photogenerated holes (h^+^), hydroxyl radicals (·OH), and superoxide radicals (·O_2_^−^).

Based on the identified intermediates and their corresponding mass-to-charge ratios, plausible degradation pathways of SMX under the action of h^+^, ·OH, and ·O_2_^−^ were proposed, as depicted in Figure 5f. The degradation of SMX proceeds mainly through three parallel pathways involving hydroxylation, amino oxidation, S–N bond cleavage, and isoxazole ring opening. In pathway I, SMX is initially attacked by ·OH (or h^+^), leading to the formation of a hydroxylated intermediate P1. Subsequent oxidation by ·O_2_^−^ induces bond cleavage, generating two smaller fragments: an aromatic structure containing a hydroxylated sulfonyl group (P2) and a fragment (P3) derived from the cleavage of the isoxazole moiety. In pathway II, ·O_2_^−^ plays a dominant role in the initial oxidation of SMX. Through electron transfer processes, oxidation and substitution reactions occur on the aromatic ring, yielding intermediate P4. The combined action of ·OH and ·O_2_^−^ further promotes SMX bond cleavage, producing intermediates P5 and P6. In pathway III, SMX degradation is primarily driven by ·OH attack on the isoxazole ring. Initial hydroxylation leads to the formation of P7, followed by continued ·OH oxidation that causes S–N bond cleavage, yielding P8. With further exposure to ·OH and ·O_2_^−^, the isoxazole ring opens to form low-molecular-weight nitrogen-containing intermediates (P10), which are subsequently oxidized to P11.

The toxicity parameters of the degradation products were evaluated using the T.E.S.T. toxicity estimation software Version 5.1.2 (Figure 5g). The results indicate that most degradation products exhibit substantially lower mutagenicity, developmental toxicity, bioaccumulation potential, and acute toxicity to fathead minnow (96 h LC_50_) compared with SMX. These findings indicate that the use of a performance-optimized PFC system combined with advanced photoanode for sulfamethoxazole treatment can effectively achieve the low-toxicity conversion of sulfamethoxazole.

## 4. Conclusions

Overall, this study elucidates the synergistic effect between the microscopic internal electric field and the macroscopic polarization in the PFC, which significantly improves the degradation efficiency of SMX by the PFC. The BTNA-2, which has abundant oxygen vacancies, has a nearly four times higher carrier separation efficiency compared to the unmodified TiO_2_. The formed Ti-O-Bi interface path and internal electric field effectively inhibit electron–hole recombination, prolong the carrier lifetime, and enhance the photocatalytic activity and power generation efficiency. This system can efficiently degrade SMX under simulated sunlight, mainly driven by h^+^, ·OH, and ·O_2_^−^ reactions. Through LC-MS and DFT, the key degradation pathways—hydroxylation, ring cleavage, and bond breaking—were clarified. Crucially, toxicity assessment confirmed that the ecological toxicity risk of the degradation intermediate products is significantly lower than that of SMX. Compared with previously reported PFC systems for antibiotic degradation, the present study emphasizes the mechanistic clarification of electric field-driven charge dynamics. This work provides mechanistic insights into interfacial charge regulation for enhanced photocatalytic degradation in PFC systems. These findings indicate that creating an internal electric field and adjusting the intrinsic electronic structure of the photocatalyst through interface engineering is crucial for advancing high-performance PFC technologies.

## Data Availability

Data are contained within the article and Supplementary Materials.

## Supplementary Materials

The following supporting information can be downloaded at: https://www.mdpi.com/article/10.3390/nano16060354/s1, Text S1. Internal electric field (IEF) intensity. Text S2. Charge separation efficiency. Text S3. The calculation of depletion width (Wb). Text S4. First-principles calculations. Text S5. Quantum chemistry calculations. Figure S1. SEM of (a) BTNA-1, (b) BTNA-2, (c) BTNA-3 and EDS elemental mapping of BTNA-2 (d). Figure S2. Survey XPS spectra of TNA and BTNA-2. Figure S3. The i-t curves of TNA and BTNA-2 photoanodes in different electrolytes. Figure S4. Mott–Schottky plot of TNA and BTNA-2 photoanodes. Figure S5. The OCP attenuation curves of TNA and BTNA-2 photoanodes. Figure S6. EIS spectra of TNA and BTNA-2: high-frequency region (a) and low-frequency region (b). Bode plot of TNA (c) and BTNA-2 (d). Figure S7. Surface states capacitance of TNA and BTNA-2 photoanodes. Figure S8. LSV curves of TNA and BTNA-2 photoanodes. Figure S9. CV curves of TNA photoanode. Figure S10. The recycling and degradation experiment of BTNA-2. Table S1. Condensed Fukui function calculation results. Refs. [24,25,32,33,34,35,36,37] are cited in the supplementary materials.
