# Supplementary material for: Synergistic Coupling of Intrinsic Internal Electric Field and Macroscopic Polarization in a Photocatalytic Fuel Cell for Efficient Antibiotic Degradation
Source: Nanomaterials (Basel). 2026 Mar 13;16(6):354. doi: 10.3390/nano16060354 (PMC13029594; doi:10.3390/nano16060354)
Supplement: Supplementary file 1 [file nanomaterials-16-00354-s001.zip › nanomaterials-4172089-supplementary.pdf]

Support Information

Synergistic Coupling of Intrinsic Internal Electric Field and Macroscopic Polarization in a Photocatalytic Fuel Cell for Efficient Antibiotic Degradation

Xicheng Li [1], Bicheng Ji [1], Jiajie Bao [1], Jiuwei Wu [2,*] and Changzheng Wang [1,3,*]

1 Institute of Advanced Materials, Beijing Key Laboratory of Functional Materials for Building Structure and Environment Remediation, School of Environment and Energy Engineering, Beijing University of Civil Engineering and Architecture, Beijing 100044, China;

lecter_lee@163.com (X.L.); bichengji@163.com (B.J.); baojiajie2505@163.com (J.B.)

2 Department of Nuclear Technology and Application, China Institute of Atomic Energy, Beijing 102413, China

3 Engineering Practice Innovation Center, Beijing University of Civil Engineering and Architecture, Beijing 102616, China

* Correspondence: jaywoo@139.com (J.W.); wcz@bucea.edu.cn (C.W.)

Supplementary Text

Text S1: Internal electric field （IEF） intensity

According to Le Formal and Grätzel, the internal electric field is proportional to the surface accumulated charge, obtained by integrating the transient photocurrent density minus its steady-state value over time. The IEF of the catalyst can be calculated based on Kanata et al. [1–3]:

$$E = (\frac{-2V_s\rho}{\varepsilon\varepsilon_0})^{\frac{1}{2}} \qquad \text{Eq.S1}$$

where E, V_s , ρ , ε and ε_0 represent the IEF magnitude, surface potential, surface charge density, low-frequency dielectric constant, and vacuum permittivity, respectively.

Text S2: Charge separation efficiency

To measure the photogenerated charge separation efficiency, 0.1 M Na_2SO_3 was used as both the electrolyte and hole scavenger. The separation efficiency (η) was calculated as follows:

$$\eta = J_{SO_4^{2-}}/J_{SO_3^{2-}} \qquad \text{Eq.S2}$$

where $J_{SO_4^{2-}}$ and $J_{SO_3^{2-}}$ are the photocurrent densities measured under different electrolyte conditions [4].

Text S3: The calculation of depletion width (W_b)

The depletion width depends on the band bending in the semiconductor's depletion region and can be calculated using the following equation:

$$W_b = \sqrt{\frac{2\varepsilon\varepsilon_0 V_{FB}}{qN_D}} = \sqrt{\frac{2\varepsilon\varepsilon_0(E_{OC}-E_{FB})}{qN_D}} \qquad \text{Eq.S3}$$

where q is the elementary charge, ε is the dielectric constant of the semiconductor, ε_0 is the vacuum permittivity, N_D is the carrier concentration, E_{OC} is the open-circuit potential at dark equilibrium, and E_{FB} represents the flat-band potential [5].

Text S4: First-principles calculations

The Perdew–Burke–Ernzerhof (PBE) generalization of the Generalized Gradient

Approximation (GGA) was employed to address electron exchange and correlation interactions in this analysis [6]. A plane-wave basis set is employed in the calculations, with a truncation energy of 490 eV chosen to ensure computational accuracy. The ionic relaxation of the system is optimized through geometry optimization, with a convergence criterion for the force set at EDIFFG = -0.03 eV/Å, indicating that the optimization process halts only when the force acting on all atoms falls below 0.03 eV/Å. All calculations are performed on a fully relaxed lattice structure, ensuring the reliability and accuracy of the results.

Text S5 Quantum chemistry calculations

The regioselectivity of ROS attacks on TCH molecules is explained based on the Fukui index. Specifically, the Fukui function plays an important role in DFT calculation and is widely used to predict reaction sites for electrophilic, nucleophilic, and radical attacks. The Fukui function was defined as

$$f(r) = \left[\frac{\partial \rho(r)}{\partial N}\right]_v \quad \text{Eq. S4}$$

where ρ(r) is the electron density at a point r in space, N is the electron number in the present system, and the constant term v in the partial derivative is external potential.[2] In the condensed version of the Fukui function, the atomic population number was used to represent the amount of electron density distribution around an atom. The condensed Fukui function could be calculated as

$$\text{Nucleophilic attack: } f_k^+ = q_N^k - q_{N+1}^k \quad \text{Eq. S5}$$

$$\text{Electrophilic attack: } f_k^- = q_{N-1}^k - q_N^k \quad \text{Eq. S6}$$

$$\text{Radical attack: } f_k^0 = (q_{N-1}^k - q_{N+1}^k)/2 \quad \text{Eq. S7}$$

where q^k is the atom charge of atom K at the corresponding state, and the values of the Fukui index of the reactive sites were usually larger than other regions.

In this study, the isosurfaces and isopotential maps of the Fukui function, frontier molecular orbital and average local ionization energy on the surface of the TCH molecule were visualized by using Multiwfn in combination with VMD [7,8].

Supplementary Figures

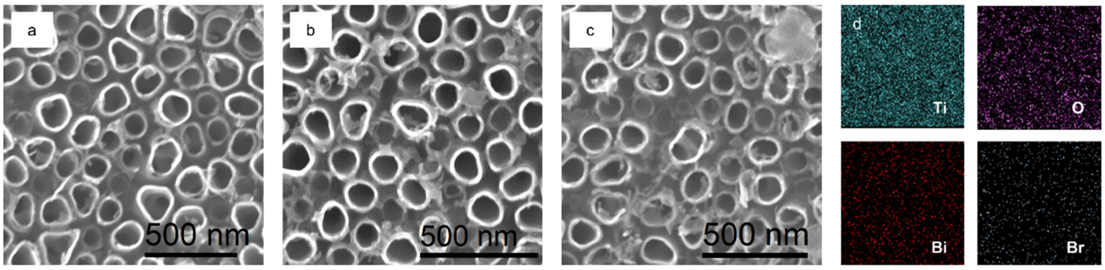

Figure S1. SEM of (a) BTNA-1, (b) BTNA-2, (c) BTNA-3 and EDS elemental mapping of BTNA-2(d).

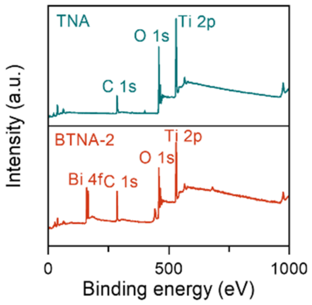

Figure S2. Survey XPS spectra of TNA and BTNA-2.

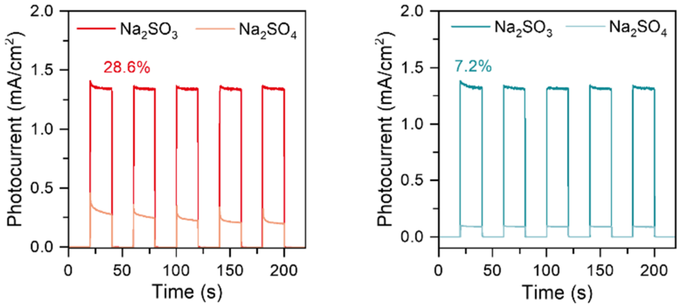

Figure S3. The i-t curves of TNA and BTNA-2 photoanodes in different electrolytes.

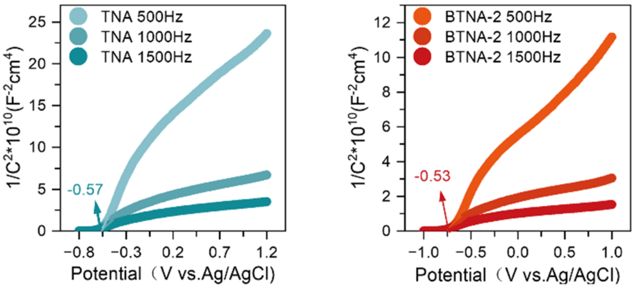

Figure S4. Mott–Schottky plot of TNA and BTNA-2 photoanodes.

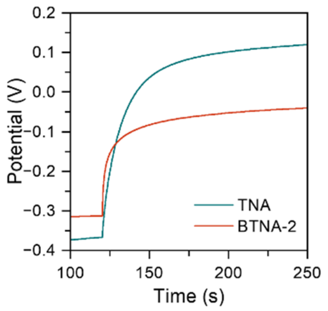

Figure S5. The OCP attenuation curves of TNA and BTNA-2 photoanodes.

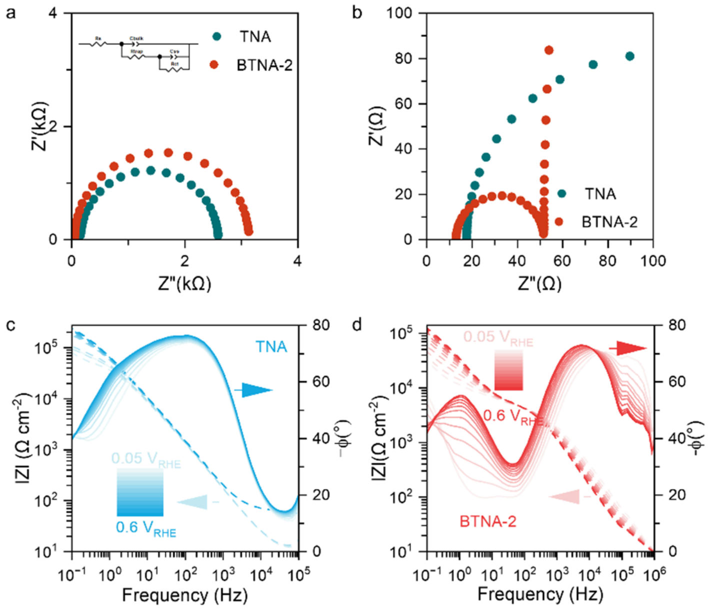

Figure S6. EIS spectra of TNA and BTNA-2: high-frequency region (a) and low-frequency region (b). Bode plot of TNA (c) and BTNA-2 (d).

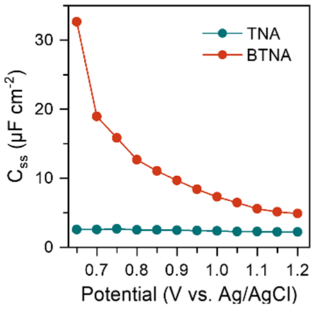

Figure S7. Surface states capacitance of TNA and BTNA-2 photoanodes.

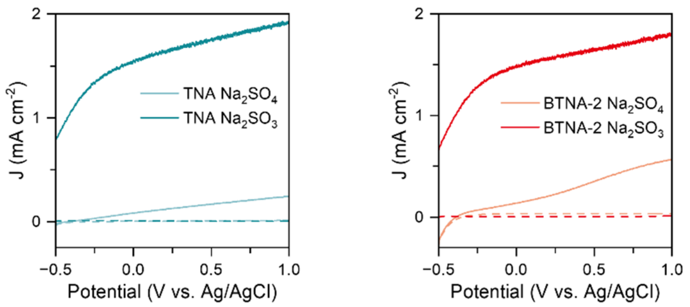

Figure S8. LSV curves of TNA and BTNA-2 photoanodes.

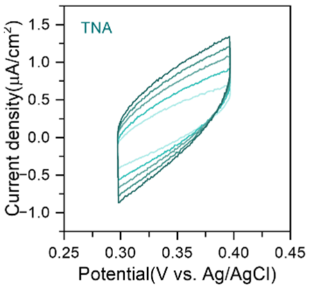

Figure S9. CV curves of TNA photoanode.

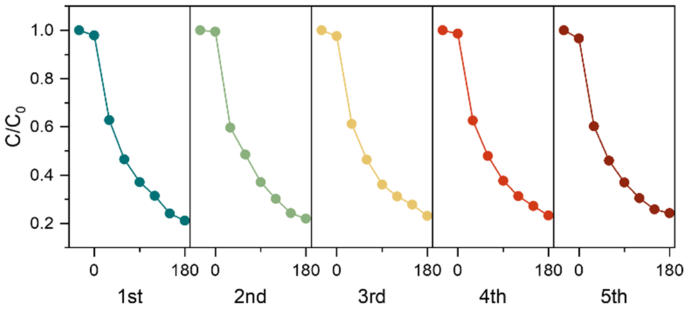

Figure S10. The recycling and degradation experiment of BTNA-2

Supplementary Table

Table S1 Condensed Fukui function calculation results.

Atom	No.	q(N)	q(N+1)	f^-	f^0
C	1	-0.0572	-0.0915	0.0689	0.0516
C	2	-0.0244	-0.0642	0.0439	0.0418
C	3	-0.046	-0.0765	0.0886	0.0595
C	4	-0.0271	-0.0598	0.0437	0.0382
C	5	-0.0591	-0.0983	0.0734	0.0563
C	6	0.0602	0.0128	0.0613	0.0543
H	7	0.0426	-0.0268	0.0374	0.0534
H	8	0.0509	0.0181	0.0315	0.0322
H	9	0.0487	0.0178	0.0314	0.0312
H	10	0.0422	-0.0307	0.0383	0.0556
N	11	-0.1568	-0.2039	0.1451	0.0961
H	12	0.1211	-0.0056	0.0551	0.0909
H	13	0.1211	-0.0016	0.0553	0.089
S	14	0.5078	0.4755	0.0234	0.0279
O	15	-0.332	-0.3657	0.0549	0.0443
O	16	-0.3325	-0.3641	0.0535	0.0425
N	17	-0.2009	-0.2309	0.0412	0.0357
H	18	0.1215	0.0516	0.0283	0.0491
H	19	0.1208	0.0543	0.0243	0.0454

References

[1] P. Lefebvre, J. Allègre, B. Gil, H. Mathieu, N. Grandjean, M. Leroux, J. Massies, P. Bigenwald, Time-resolved photoluminescence as a probe of internal electric fields in GaN-(GaAl)N quantum wells, Phys. Rev. B 59 (1999) 15363–15367.

[2] G. Morello, F. Della Sala, L. Carbone, L. Manna, G. Maruccio, R. Cingolani, M. De Giorgi, Intrinsic optical nonlinearity in colloidal seeded grown CdSe/CdS nanostructures: Photoinduced screening of the internal electric field, Phys. Rev. B 78 (2008) 195313.

[3] J. Seo Im, H. Kollmer, J. Off, A. Sohmer, F. Scholz, A. Hangleiter, Reduction of oscillator strength due to piezoelectric fields in GaN/AlxGa1-xN quantum wells, Phys. Rev. B 57 (1998) R9435–R9438.

[4] L. Liu, J. Hu, Z. Ma, Z. Zhu, B. He, F. Chen, Y. Lu, R. Xu, Y. Zhang, T. Ma, M. Sui, H. Huang, One-dimensional single atom arrays on ferroelectric nanosheets for enhanced CO2 photoreduction, Nat. Commun. 15 (2024) 305.

[5] C. Li, A. Li, Z. Luo, J. Zhang, X. Chang, Z. Huang, T. Wang, J. Gong, Surviving High‐Temperature Calcination: ZrO2-Induced Hematite Nanotubes for Photoelectrochemical Water Oxidation, Angew. Chem. Int. Ed. 56 (2017) 4150–4155.

[6] Seifert, G., Porezag, D. and Frauenheim, T. Calculations of molecules, clusters, and solids with a simplified LCAO-DFT-LDA scheme. Int. J. Quantum Chem., 58(1996) 185-192.

[7] T. Lu, F. Chen, Multiwfn: A multifunctional wavefunction analyzer, J. Comput. Chem. 33 (2012) 580–592.

[8] T. Lu, A comprehensive electron wavefunction analysis toolbox for chemists, Multiwfn, The J. Chem. Phys. 161 (2024) 082503.
